# NLRP3 Inflammasome as a Molecular Marker in Diabetic Cardiomyopathy

**DOI:** 10.3389/fphys.2017.00519

**Published:** 2017-07-25

**Authors:** Beibei Luo, Feng Huang, Yanli Liu, Yiying Liang, Zhe Wei, Honghong Ke, Zhiyu Zeng, Weiqiang Huang, Yan He

**Affiliations:** ^1^Department of Geriatric Cardiology, The First Affiliated Hospital of Guangxi Medical University Nanning, China; ^2^Department of Cardiology, Institute of Cardiovascular Diseases, The First Affiliated Hospital of Guangxi Medical University Nanning, China

**Keywords:** NLRP3 inflammasome, thioredoxin interacting/inhibiting protein, pyroptosis, inflammation, diabetic cardiomyopathy

## Abstract

Diabetic cardiomyopathy (DCM), a common consequence of longstanding diabetes mellitus, is initiated by death of cardiomyocyte. Hyperglycemia-induced reactive oxygen species (ROS) overproduction is a major contributor of the chronic low-grade inflammation that characterizes as the DCM. ROS may promote the activation of nucleotide-binding oligomerization domain like receptor (NLR) pyrin domain containing 3 (NLRP3) inflammasome, a novel regulator of inflammation and cell death, by nuclear factor-kB (NF-κB) and thioredoxin interacting/inhibiting protein (TXNIP). NLRP3 inflammasome regulates the death of cardiomyocyte and activation of fibroblast in DCM, which is involved in the structural and functional disorder of DCM. However, comprehensive understanding of molecular mechanisms linking NLRP3 inflammasome and disorder of cardiomyocyte and fibroblast in DCM is lacking. Here, we review the molecular mechanism(s) of NLRP3 inflammasome activation in response to hyperglycemia in DCM.

## Introduction

Diabetic cardiomyopathy (DCM), one of the severe complication of diabetes mellitus, is the leading cause of death in diabetes patients (Shaw et al., 2010). DCM is characterized by structural and functional disorders, including myocardial cell death, myocardial fibroblast activation, left ventricular dysfunction, and metabolic deregulation (Westermeier et al., 2015). Among them, the death of cardiomyocyte is thought to be the basic change of DCM, which initiates cardiac remodeling and results in left ventricular dysfunction (Kuethe et al., 2007; Liu et al., 2013).

From the standpoints of cellular death and inflammation, a newly described inflammatory mechanism fundamental to innate immune system is proposed to contribute to DCM (Luo et al., 2014a,b). In particular, the nucleotide-binding oligomerization domain like receptor (NLR) pyrin domain containing 3 (NLRP3) inflammasome, expressed abundantly in cardiomyotytes, may play important roles in the process of myocardial cell death.

A growing list of molecules has been examined as possible molecular markers for heart failure (HF) and cardiovascular disease (CVD), exemplified by the myocyte injury markers troponins I and T, the myocyte stress markers Brain natriuretic peptide (BNP) and ST2/IL-33, and the inflammation markers C-reactive protein (CRP) and certain cytokines (TNF, IL-1β, and IL-18; Braunwald, 2008). Troponin I and T are used to diagnose acute myocardial infarction and prognose thrombotic ACS. BNP and NT-pro-BNP are used to predict death or re-hospitalization for HF in a number of conditions and distinguish acute from chronic HF. ST2/IL-33 also can be used to predict cardiac damage and disease progression. CRP measurement is a useful tool for determining prognosis in HF and myocarditis patients. According to the “cytokine hypothesis,” cytokines play important roles in the pathogenesis of HF and CVD and are valuable to indicate inflammation and disease progression.

Increasing evidence reveals that NLRP3 inflammasome can be used as a promising molecular marker for future development of effective and targeted therapies with lower toxicity in DCM and other CVD. We found that the protective effect of the anti-inflammation drug rosuvastatin (RSV) was associated with its ability to inhibit the activation of NLRP3 inflammasome via TXNIP and suppress the phosphorylation of MAPK signal pathways in DCM (Luo et al., 2014a). The synthetized NLRP3 inflammasome inhibitor INF4E was also found to protect against the IR-induced myocardial injury by inhibiting NLRP3 inflammasome, leading to the activation of the prosurvival RISK pathway and improvement in mitochondrial function (Mastrocola et al., 2016b). Since previous anti-inflammatory drug therapy can lead to compromised host defense or secondary inflammatory processes due to the compensatory responses, targeting specific inflammatory pathways (such as NLRP3 inflammasome) may provide a more precise approach to reducing deleterious inflammation without affecting other innate host defense (Butts et al., 2015). Moreover, blocking early inflammatory mediators such as TXNIP (as well as NLRP3 inflammasome components) that significantly induced by hyperglycemia will represent the excellent targets to prevent diabetes and its complications DCM (Singh, 2014). In this review, we will summarize the available evidence, including the regulatory mechanism and biological function of NLRP3 inflammasome in DCM, which will elucidate the significance of NLRP3 inflamamsome as the molecular marker of DCM.

## NLRP3 inflammasome biology

NLRP3 inflammasome consists of NLRP3, apoptosis-associated speck-like protein containing a caspase recruitment domain (ASC) and pro-caspase-1 (Shao et al., 2015). NLRP3 contains three domains: a C-terminal leucine-rich repeats domain (LRRs), a central nucleotide binding and oligomerization domain (NACHT), and an N-terminal pyrin domain (PYD). ASC is composed of a C-terminal caspase recruitment domain (CARD) and an N-terminal PYD. Pro-caspase-1 is consists of a CARD, a p20 and a p10 domain (Jo et al., 2016). Once activated, the PYD of NLRP3 could bind to that of ASC, and subsequently the CARD of ASC recruits and interacts with pro-caspase-1. These interactions form the NLRP3 inflammasome and promote the autocatalytic cleavage of pro-caspase-1, which results in the production of activated caspase-1 (Lamkanfi and Dixit, 2014).

The activated caspase-1 has two major functions: the regulated function that processes pro-IL-18 and pro-IL-1β into their mature forms (IL-18 and IL-1β) and the novel function that induces pyroptosis. Accumulated evidence indicated that IL-18 and IL-1β were important proinflammatory cytokines in the development of CVD by suppressing cardiac contractility, promoting myocardial hypertrophy, and inducing cardiomyocyte apoptosis (Loppnow et al., 2001; Apostolakis et al., 2008; Qamar and Rader, 2012). Pyroptosis, an inflammatory form of programmed cell death, was dependent on caspase-1 activity (Guarda and So, 2010). The morphology of pyroptosis partially overlaps with both apoptosis and necrosis (Coll et al., 2011). Similar to apoptotic cells, pyroptotic cells incur DNA damage, TUNEL staining positive, annexin V staining positive (Miao et al., 2011). As in necrosis, pyroptosis triggers pore formation in the cell membrane, release of pro-inflammatory cytosolic content, and cell lysis. Pyroptosis has been found in response to infection with several bacteria and viruses (Allen et al., 2009; Bergsbaken et al., 2009). However, aberrant or excessive activation of the NLRP3 inflammasome contribute to development of autoimmune and even metabolic diseases, such as type 2 diabetes (Donath and Shoelson, 2011), atherosclerotic disease (Rajamaki et al., 2016; Wang et al., 2017), obesity (Ahmad et al., 2016), gouty Arthritis (Liu Y. F. et al., 2016; Table 1). Recently, we and other researchers reported that pyroptosis were involved in non-infectious disease, such as atherosclerosis and DCM (Luo et al., 2014b; Wree et al., 2014).

**Table 1:** NLRP3 inflammasome and metabolic disorders.

**Metabolic disorders**	**Activators/signals**	**Major contributors**
Atherosclerotic disease	Cholesterol crystals	IL-1β, IL-18
Type 2 diabetes	Amylin/IAPP, glucose, palmitate, and ceramide	IL-1β
Obesity	High fat diet induces cholesterol crystal formation	IL-1β, IL-18
Gouty arthritis	Monosodium urate (MSU)	IL-1β

## The activation of NLRP3 inflammasome in DCM

A diverse set of pathogen- and host-derived ligands can activate the NLRP3 inflammasome. These include pathogen-associated molecular patterns (PAMPs), bacterial pore-forming toxins, hemozoin, silica, asbestos, UV light, ATP, glucose, MSU, calcium pyrophosphate dehydrate, amyloid β, hyaluronan, alum, danger-associated molecular patterns (DAMPs), and environmental stimuli (Schroder et al., 2010). The stimuli of NLRP3 inflammasome were classically seen to converge on three distinct signal pathways: (1) NLRP3 senses potassium efflux and loss of membrane integrity through purinergic receptor P2X7 and pannexin-1, respectively; (2) crystalline or particulate-accelerated lysosomal destabilization promote cathepsin B to direct NLRP3 ligands; (3) DAMP/PAMP-induced reactive oxygen species (ROS) trigger the activation of NLRP3 inflammasome (Zhou et al., 2010). Recent researches have indicated various other activating mechanisms of NLRP3 inflammasome. Among these, mitochondrial dysfunction was thought to be the pivotal step for the NLRP3 inflammasome activation induced by NLRP3 agonists. Mitochondria can promote NLRP3 inflammasome activation through mitochondrial-derived signals, such as mROS, mtDNA, and cadiolipin (Zhou et al., 2010; Nakahira et al., 2011; Iyer et al., 2013). Besides mitochondria, ER-stress, guanylate-binding protein 5 (GBP5), and double-stranded RNA-dependent protein kinase (PKR) were also shown to be correlated with NLRP3 inflammasome activation (Lerner et al., 2012; Lu et al., 2012; Shenoy et al., 2012).

During NLRP3 inflammasome activation, the agonist can induce an initial priming step (the first signal) that promotes NLRP3 expression, followed by the structural modulation step (the second signal) that induces NLRP3 inflammasome assembly. Nuclear factor-kB (NF-κB), which controls the transcriptional induction of *NLRP3*, was shown to provide the first signal in the activation of NLRP3 inflammasome (Corsini et al., 2013; Liao et al., 2013; Luo et al., 2014b). Thioredoxin interacting/inhibiting protein (TXNIP), deubiquitination, and oxidized mitochondrial DNA were suggested to provide the second signal by binding with NLRP3 directly and modulating its oligomerization (Zhou et al., 2010; Luo et al., 2014b). Selected DAMPs associated with atherosclerosis are found to provide both signals for inflammasome activation (Robbins et al., 2014).

Type 1 diabetes, resulting from the autoimmune destruction of insulin-producing β cells in the pancreas, is characterized by low level of insulin and hyperglycemia (Nakayama et al., 2015). Most diabetes patients have type 2 diabetes, which is characterized by hyperglycemia, hyperlipidemia, and insulin resistance (Pandey et al., 2015). Several studies have indicated that NLRP3 inflammasome expression was increased in circumstance of glycotoxicity and lipotoxicity (Zhou et al., 2010; Vandanmagsar et al., 2011). Furthermore, NLRP3 inflammsome could be activated by saturated fatty acid, ceramides, modified LDL, and hyperglycemia in obesity and type 2 diabetes (Wen et al., 2011; Jin and Flavell, 2013). During the process of DCM, DAMP -induced ROS generation is the most well-studied pathway of NLRP3 inflammasome activation. Our previous studies have addressed the role of NLRP3 inflammasome in DCM using the rat model of T2DM (Luo et al., 2014a,b). High glucose-mediated ROS generation could upregulate NF-κB phosphorylation and TXNIP expression, which account for NLRP3 priming and the secondary step of activation (Bryant and Fitzgerald, 2009; Franchi et al., 2012; Kumar et al., 2013). NF-κB has been shown to increase the expression of NLRP3, pro-caspase-18 and pro-IL-1β, which facilitating the activation of NLRP3 inflammasome (Donath et al., 2010; Qiao et al., 2012; Boaru et al., 2015). TXNIP has been reported to be another essential link between ROS and NLRP3 inflammasome by priming the expression of NLRP3 inflammasome or modulating the structure of NLRP3 directly (Martinon, 2010).

Lipotoxicity occurring in type 2 diabetes plays a non-negligible role on inflammasome assembly. Free fatty acid might induce the activation of NLRP3 inflammasome by ROS production and ER stress (Legrand-Poels et al., 2014). And intramyocellular lipid accumulation in cardiomyocytes has been frequently reported in diet-induced diabetes (Wolf et al., 2016; Zlobine et al., 2016). However, there was no evidence that intramyocellular lipid could promote the activation of intercellular NLRP3 inflammasome directly.

## NLRP3 inflammasome-mediated inflammation in DCM

Glucose has been reported to be one of the effective activators of NLRP3 inflammasome (Shi H. et al., 2015; Zu et al., 2015). Recent data also suggest that NLRP3 is responsible for the cardiac inflammation of glycotoxicity during the process of T2DM and DCM (Vandanmagsar et al., 2011; Luo et al., 2014b). IL-1β and IL-18 are the main effectors of ROS-mediated NLRP3 inflammasome activation in DCM. IL-1β and IL-18 have a central role in the cardiomyocyte apoptosis and fibroblast activation of DCM, which is thought to be the initiator of the structural disorder (Santiago et al., 2014; Somanna et al., 2015). NLRP3 inflammasome-mediated pyroptosis in DCM.

Pyroptosis is a caspase-1- or caspase-11-dependent cell death process, which is characterized by plasma membrane pore formation, cell swelling, cell osmotic lysis, and release of proinflammastory intracellular contents (de Zoete et al., 2014; Shalini et al., 2015). Pyroptosis was firstly reported in the research of the infection with *Shigell flexneri* and *Salmonell typhimurium*, which was considered to be key responses of immune response to pathogens (Zychlinsky et al., 1992; Monack et al., 1996). Subsequent studies discovered that pyroptosis also occurred in cells induced by non-infectious stimuli (Jang et al., 2015; Lebeaupin et al., 2015; Lopez-Pastrana et al., 2015). Within the type 2 diabetes rat model and H9c2 cardiomyoctye cell line, our previous work showed the important characteristics of pyroptosis in myocardium and cardiomyocyte, including activated caspase-1, cytoplasmic swelling, and nucleus DNA damage. NLRP3-siRNA lentivirus treatment, followed by reduced activation of caspase-1, abrogated the pyroptosis both in the myocardium of DCM and the high glucose-treated cardiomyocyte. NLRP3 inflammasome turned out to be a pivotal regulator of caspase-1-depended pyroptosis in the pathogenesis of DCM.

Not much research has been done to explore the key molecules involved in caspase-1-induced pyroptosis. Recent studies identified that the protein gasdermin D (GSDMD), encoded by a gene named *Gsdmd*, was required and sufficient for pyroptosis in mouse and human cells (He et al., 2015; Kayagaki et al., 2015; Shi J. et al., 2015; Shi et al., 2017). Mechanistically, caspase-1 and other inflammatory caspases (caspase-4/5/11) cleave GSDMD into two fragments, and the resulting amino-terminal fragment promotes pyroptosis by its pore-forming activity to rupture the membrane (Ding et al., 2016; Liu X. et al., 2016). Given the similar pore-forming activity in different gasdermins, the concept of pyroptosis is thus redefined as gasdermin-mediated programmed necrotic cell death (Shi et al., 2017). Other pyroptosis-inducing caspase-1 substrates still await to be discovered.

## NLRP3 inflammasome-promoted fibrosis in DCM

Our previous work showed that NLRP3-siRNA lentivirus treatment reduced the aberrant expression of collage I and III in myocardium of DCM rat model (Luo et al., 2014b). Other research revealed that NLRP3 inflammasome suppressed the cAMP expression of cardiac fibroblast in mice with sepsis, which inhibited the cardiac contraction (Zhang et al., 2014). With TGF-β stimulation, NLRP3 inflammasome increased in cardiac fibroblast, which facilitated the myofibroblast and receptor associated Smad (R-Smad) activation. The activated R-Smad could associate with co-Smad to form the transcription complex that promotes the expression of profibrotic gene (Bracey et al., 2014). Whether the R-Smad is the pivotal link between NLRP3 inflammasome and high glucose-induced cardiac fibroblast activation remains unknown.

## Conclusions

NLRP3 inflammasome is a multiprotein signaling complex of the innate immune system essential for controlling the inflammatory response and coordinating antimicrobial host defense. Hyperglycemia-induced ROS overproduction is a major contributor of chronic low-grade inflammation. Therefore, NLRP3-dependent pyroptosis and maturation of pro-inflammatory effectors (IL-1β/IL-18) induced by ROS could contribute to development of autoimmune and even metabolic diseases, such as DCM.

**Figure 1:**
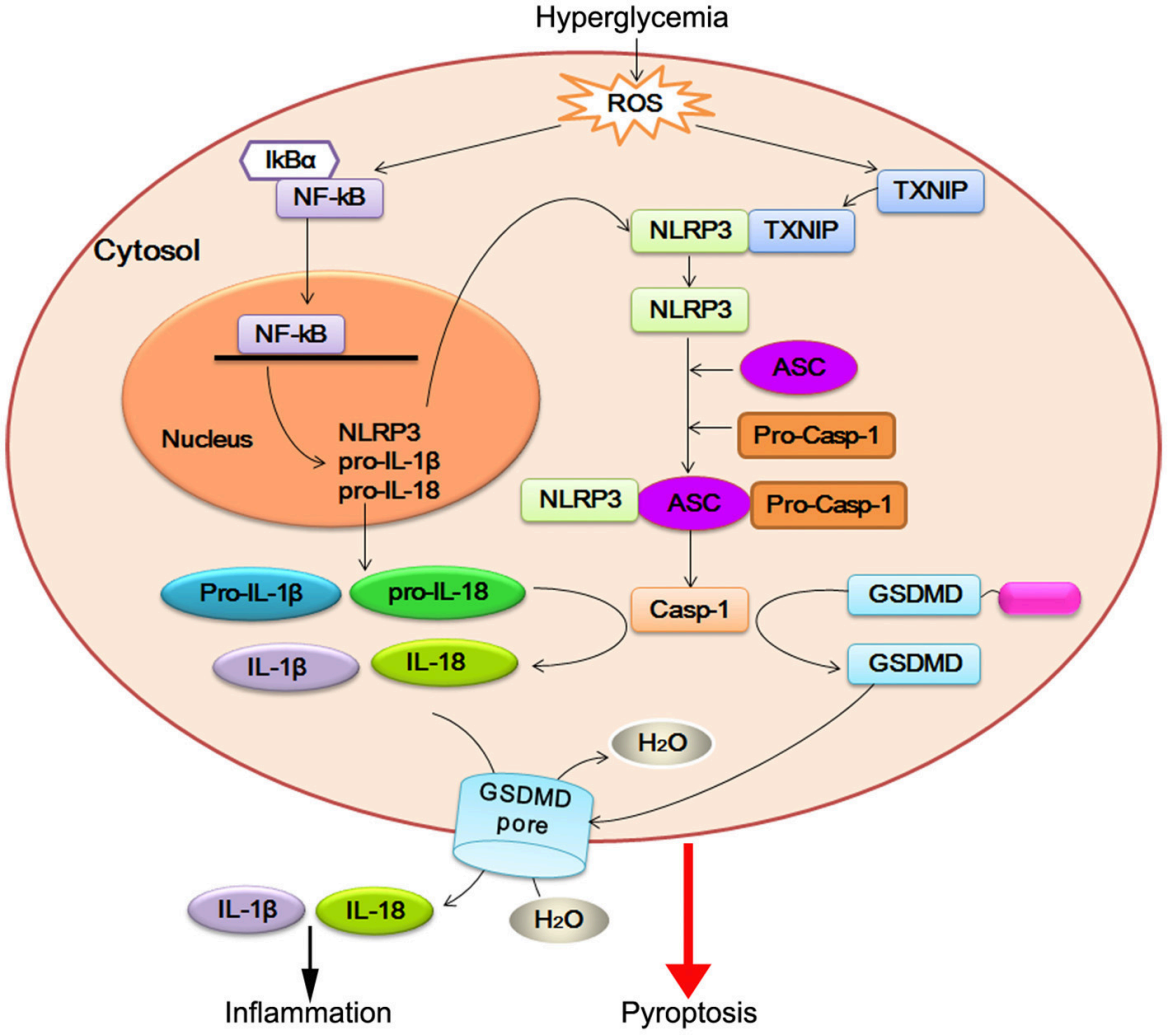
NLRP3 inflammasome activation in DCM. Hyperglycemia-induced reactive oxygen species (ROS) leads to nuclear factor-kB (NF-κB) and TXNIP overexpression. NF-κB increases the expression of NLRP3, pro-IL-18, and pro-IL-1β. TXNIP modulates the biological structure of NLRP3 leading to NLRP3 inflammasome assembly and pro-caspase-1 (pro-casp-1) autocleavage. Active caspase-1 (Casp-1) promotes pro-IL-18 and pro-IL-1β maturation, which facilitate inflammatory reaction. On the other hand, active caspase-1 cleaves GSDMD within the linker between its N-terminal (blue) and C-terminal (magenta). The released GSDMD-N domain oligomerizes to generate membrane pores, which disrupts the osmotic potential and leads to cell swelling and eventual lysis.

Identification of NLRP3 inflammasome platforms is the main breakthrough on DCM research, as it is in the area of HF (Butts et al., 2015) and other cardiometabolic diseases (Janket et al., 2015; Mastrocola et al., 2016a). ROS-dependent NF-κB and TXNIP appear to regulate both priming and post-translational steps in the activation of NLRP3 inflammasome. After NLRP3 inflammasome activation, caspase-1 promotes a novel programmed cell death process, named pyroptosis, in cardiomyocyte of DCM. Pro-inflammatory cytokines IL-1β and IL-18 are direct substrates of caspase-1. Caspase-1 is activated by the NLRP3 inflammasome, in which a central platform, consisted of NLRP3, ASC1, and caspase1, recognizes an unknown signal or ligand. Active caspase-1 then cleaves and maturates of these cytokines and triggers pyroptosis. Together with this observation, caspase-1-mediated IL-1β and IL-18 activation initiate apoptosis of cardiomyocyte and activation of cardiofibroblast. On the other hand, NLRP3 inflammasome can induce fibrosis in DCM (Figure 1). Further investigations on the mechanism underlying caspase-1-regulated pyroptosis and IL-18/IL-1β-regulated fibroblast disorder are required to elucidate the function of NLRP3 inflammasome in DCM.

**Figure 2:**
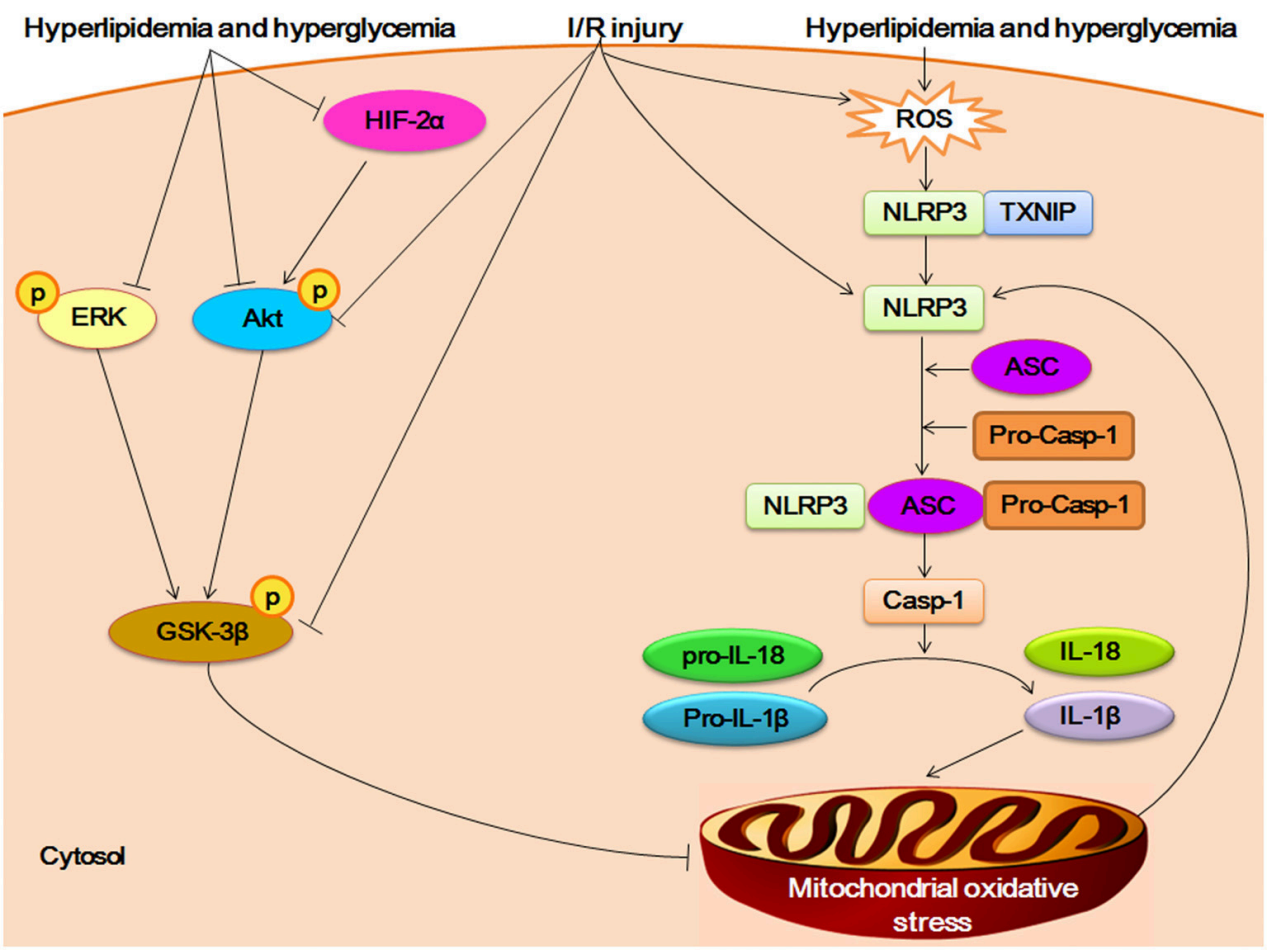
Crosstalk between NLRP3 inflammasome and RISK/HIF-2α in myocardial I/R injury. The HFHF diet induced-hyperlipidemia and hyperglycemia promote overexpression of ROS and then triggers the assembly of NLRP3 inflamamsome. The activation of NLRP3 inflammasome increase the secretion of IL-1β and IL-18. The proinflammatory cytokines accelerate the oxidative stress of mitochondrial, which in turn promotes the activation of NlRP3 inflammasome. Hyperlipidemia and hyperglycemia inhibit the phosphorylation of Reperfusion Injury Salvage Kinases (RISK) pathway (including AKT, ERK, and GSK-3β), and negatively impacts hypoxia inducible factor-2α (HIF-2α), which can worsen the mitochondrial oxidative unbalance. The I/R challenge can suppress the phosphorylation of AKT and GSK-3β, and induce the expression of ROS and activation of NLRP3.

As in DCM, NLRP3 inflammasome also plays an important role in myocardial ischemia/reperfusion (I/R) injury after high-fat high-fructose (HFHF) diet, which is a common type of cardiometabolic disease (Mastrocola et al., 2016a). The HFHF diet mouse showed excessive intake of fatty acids and sugars in cardiomyocyte, which induced the overexpression of ROS and then triggered the activation of NLRP3 inflamamsome. The upregulation of NLRP3 inflammasome enhanced the susceptibility to (I/R) injury by promoting the mitochondrial disorder via IL-1β and IL-18. On the other hand, the protective pathways of Reperfusion Injury Salvage Kinases (RISK) pathway (including AKT, ERK, and GSK-3β) and hypoxia inducible factor-2α (HIF-2α) was inhibited by fatty acids and sugar. The impairment of RISK/HIF-2α pathway can worsen the mitochondrial oxidative stress regulated by NLRP3 inflammasome, which increase the susceptibility to I/R injury in HFHF diet mouse (Figure 2).

As mentioned above, molecular markers are important tools for clinical management in CVD, facilitating in early detection of disease, diagnostic, monitor of disease state, assessment of disease risk, determination of therapy, and evaluation of therapeutic activity (Braunwald, 2008). Inflammation associated markers (CRP, cytokines and the NLRP3 inflammasome) are important in that they are also risk factors directly involved in the pathogenesis of CVD. Of these, the NLRP3 inflammasome is unique in that it provides a mechanistic explanation of cytokine activation which leads to disease progression (Luo et al., 2014a; Mastrocola et al., 2016b). Therefore, understanding the molecular mechanism of the NLRP3 inflammasome activation and targeting specifically the NLRP3 inflammasome and its interacting counterparts (e.g., TXNIP) will be of great value in clinical management of DCM and other CVD (Singh, 2014; Butts et al., 2015).

## Author contributions

BL and ZZ wrote the manuscript. FH, YaL, and YiL revised the language of the manuscript. ZW and HK prepared the figure and figure legend. BL, WH, and YH conceived the review.

### Conflict of interest statement

The authors declare that the research was conducted in the absence of any commercial or financial relationships that could be construed as a potential conflict of interest.
